# Study on a Quaternary Working Pair of CaCl_2_-LiNO_3_-KNO_3_/H_2_O for an Absorption Refrigeration Cycle

**DOI:** 10.3390/e21060546

**Published:** 2019-05-29

**Authors:** Yiqun Li, Na Li, Chunhuan Luo, Qingquan Su

**Affiliations:** 1 School of Energy and Environmental Engineering, University of Science and Technology Beijing, Beijing 100083, China; 2 State Grid Energy Conservation Service CO., Ltd., Beijing 100083, China; 3 Beijing Higher Institution Engineering Research Center of Energy Conservation and Environmental Protection, University of Science and Technology Beijing, Beijing 100083, China

**Keywords:** absorption refrigeration, working pair, crystallization temperature, vapor pressure, COP, corrosivity

## Abstract

When compared with LiBr/H_2_O, an absorption refrigeration cycle using CaCl_2_/H_2_O as the working pair needs a lower driving heat source temperature, that is, CaCl_2_/H_2_O has a better refrigeration characteristic. However, the crystallization temperature of CaCl_2_/H_2_O solution is too high and its absorption ability is not high enough to achieve an evaporation temperature of 5 °C or lower. CaCl_2_-LiNO_3_-KNO_3_(15.5:5:1)/H_2_O was proposed and its crystallization temperature, saturated vapor pressure, density, viscosity, specific heat capacity, specific entropy, and specific enthalpy were measured to retain the refrigeration characteristic of CaCl_2_/H_2_O and solve its problems. Under the same conditions, the generation temperature for an absorption refrigeration cycle with CaCl_2_-LiNO_3_-KNO_3_(15.5:5:1)/H_2_O was 7.0 °C lower than that with LiBr/H_2_O. Moreover, the cycle’s COP and exergy efficiency with CaCl_2_-LiNO_3_-KNO_3_(15.5:5:1)/H_2_O were approximately 0.04 and 0.06 higher than those with LiBr/H_2_O, respectively. The corrosion rates of carbon steel and copper for the proposed working pair were 14.31 μm∙y^−1^ and 2.04 μm∙y^−1^ at 80 °C and pH 9.7, respectively, which were low enough for engineering applications.

## 1. Introduction

Absorption refrigeration systems can effectively utilize not only industrial waste heat [1,2,3,4], but also low-grade renewable energy, including solar energy and geothermal energy for refrigeration [5,6,7]. As a traditional working pair, LiBr/H_2_O has been widely used for refrigeration [8,9,10,11,12]. However, studies on new working pairs are still ongoing, because the required temperature of driving heat source for a refrigeration cycle using LiBr/H_2_O even reaches 88.0 °C [13,14,15], which is too high to use for some low-grade heat sources. Lin et al. [16] studied a double-stage air-cooled NH_3_/H_2_O absorption refrigeration system and found that it could effectively lower the temperature of driving heat source for utilizing solar energy. Malinina et al. [17] analyzed the influences of temperature and humidity on a solar energy refrigeration system with LiBr/H_2_O and calculated the minimum heat-collecting temperatures that are based on solar energy in some cities. Mortazavi et al. [18] designed an absorption refrigeration system with a falling-film generator, which could use lower temperature waste heat or solar energy. Bourouis et al. [19] analyzed the performance of LiBr + LiNO_3_ + LiCl + LiI + H_2_O in a vertical tube and found that the crystallization temperature was 35 °C lower than LiBr solution. Sun et al. [20] studied LiBr-LiNO_3_ (mole ratio: 4:1)/H_2_O and found the alternative working pair had higher COP and less corrisivity than LiBr/H_2_O. Chen et al. [21] studied the performance of an absorption refrigeration system using [emim]Cu_2_Cl_5_/NH_3_ as working pair with the UNIFAC model, and results showed that the [emim]Cu_2_Cl_5_/NH_3_ system possessed several advantages, including non-crystallization and non-corrosion. Bellos et al. [22] compared the exergy efficiency between LiCl/H_2_O and LiBr/H_2_O, results showed that LiCl/H_2_O performed better at different ambient temperature levels. Wang et al. [23] measured the properties of different ammonia/ionic liquid working pairs. Luo et al. [24,25,26,27,28] studied various lithium nitrate-ionic liquid/water working pairs. They both found that the working pairs with ionic liquid had excellent characteristics for heating, whereas they were not suitable for refrigeration because of insufficient absorption ability. Li et al. [29,30,31,32] measured the thermophysical properties of several CaCl_2_-based working pairs, and found that the CaCl_2_-based working pairs had an excellent refrigeration characteristic. However, their strong corrosivity limited the practical applications.

In this work, to find a new working pair with excellent refrigeration characteristic for absorption refrigeration, various inorganic salts, including NaCl, KCl, LiCl, KNO_3_, and LiNO_3_, were added in CaCl_2_/H_2_O, and their crystallization temperature and saturated vapor pressure were measured. Furthermore, some other thermophysical properties and corrisivity of the proposed working pair were measured and the performance of an absorption refrigeration cycle with the proposed working pair was analyzed.

## 2. Experiments

### 2.1. Materials

Table 1 shows the purities of the reagents used in this work. Table 2 lists the detailed compositions of carbon steel and copper samples used in the corrosion experiments.

### 2.2. Apparatus and Methods

To analyze the performance of a working pair, its properties, such as crystallization temperature, saturated vapor pressure, density, viscosity, specific heat capacity, dissolution enthalpy, and corrosion rate, need to be measured.

The crystallization temperature was measured by a dynamic method in a precision thermostat (HX-3010, Bilang, Shanghai). The prepared solution was put in the thermostat at a slightly higher initial temperature. The crystallization temperature was measured by reducing the temperature by 1 °C every 12 hours until crystallization appeared in the solution.

The saturated vapor pressure was measured by a static method. The solution was poured into an autoclave that was assembled with a precision digital absolute pressure gauge (AX-110, Aoxin, Xi’an) and a Pt-100 thermocouple. The autoclave was placed in a precision oil bath (DKU-30, Jinghong, Shanghai) after vacuuming. The data of pressure gauge and thermocouple were obtained, respectively, after stabilization.

The density and viscosity were measured in a precision viscometer oil bath (SYP1003-H, Zhongxi, Beijing). Density measurement was carried out by a capillary pycnometer with a capillary diameter of approximately 1 mm. Ubbelohde capillary viscometers with different fine capillaries was used to carry out the viscosity measurement.

The specific heat capacity and dissolution enthalpy were measured by a micro reaction calorimeter (μRC, THT Co., UK). The measurement of specific heat capacity was conducted by making a 1 °C “step-change” in the measurement temperature. The dissolution enthalpy was measured by an isothermal method, with a solid addition accessory.

The corrosion rate of carbon steel and copper in the solution were measured by a weight loss method. The sample was immerged in the solution for 200 hours. The corrosion rate was calculated according to the mass change of the sample.

References [24,25,26,27,28] give the detailed procedures. All the above experiments were carried out at 101.3 kPa and 25 °C. The properties of water and LiBr/H_2_O were measured and compared with literature values to validate the above methods. In addition, three parallel experiments were carried out for each measurement to verify the reproducibility. Table 3 lists the accuracy of the instruments.

## 3. Results and Discussion

### 3.1. CaCl_2_/H_2_O

**Figure 1:**
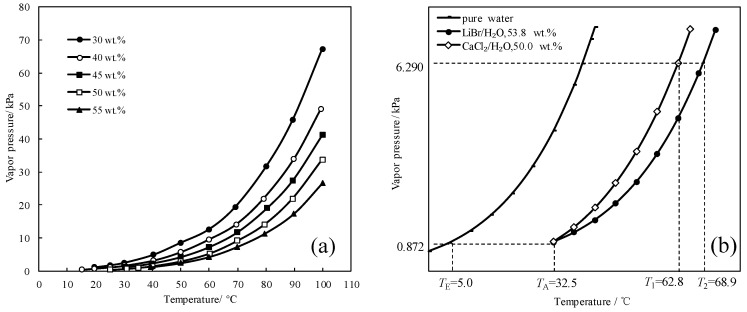
(**a**) Saturated vapor pressure of CaCl_2_/H_2_O; (**b**) Comparison of the refrigeration characteristic between LiBr/H_2_O and CaCl_2_/H_2_O.

To find a working pair with excellent refrigeration characteristic, the saturated vapor pressures of CaCl_2_/H_2_O were measured and are shown in Figure 1a. Figure 1b presents the comparison of the refrigeration characteristic between LiBr/H_2_O and CaCl_2_/H_2_O, it shows that, for an identical pressure of 6.290 kPa, which is a typical pressure in the condenser and generator, CaCl_2_/H_2_O had a lower generation temperature than LiBr/H_2_O, meaning that the refrigeration characteristic of CaCl_2_/H_2_O was better than LiBr/H_2_O.

**Figure 2:**
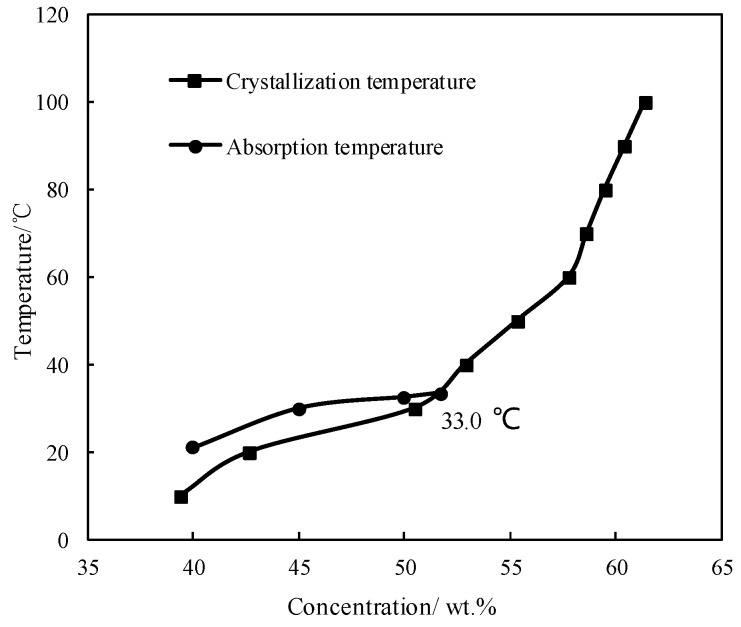
Crystallization temperature and absorption temperature for CaCl_2_/H_2_O.

Figure 2 shows the absorption temperature of CaCl_2_/H_2_O under an absorption pressure of 0.872 kPa, which corresponds to the typical evaporation temperature of 5 °C. The crystallization temperature of CaCl_2_/H_2_O is also plotted in Figure 2 to illustrate the limitation from the crystallization of absorbent. The absorption temperature increased with increasing the concentration, and meet the crystallization temperature at 33.0 °C, which was the maximum absorption temperature under the given conditions. Generally, the absorption temperature in absorber for a refrigeration cycle is 37.0 °C, so the binary working pair of CaCl_2_/H_2_O could not be applied for the refrigeration cycle, because of its high crystallization temperature and insufficient absorption ability.

To improve the absorption ability and reduce the crystallization temperature of CaCl_2_/H_2_O, some salts, including NaCl, KCl, LiCl, KNO_3_, and LiNO_3_, were combined with CaCl_2_/H_2_O, and their saturated vapor pressures and crystallization temperatures were measured.

### 3.2. Measurement of Crystallization Temperature T_C_

**Figure 3:**
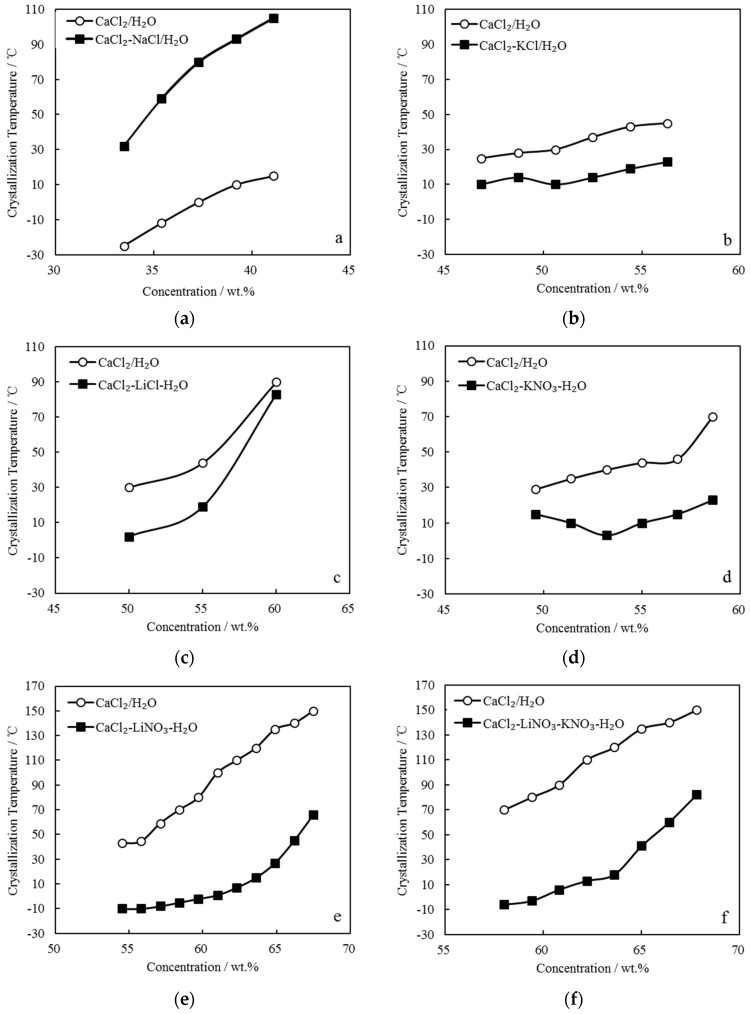
Comparison of *T_C_* between CaCl_2_/H_2_O and other CaCl_2_-based working pairs: (**a**) CaCl_2_-NaCl/H_2_O; (**b**) CaCl_2_-KCl/H_2_O; (**c**) CaCl_2_-LiCl/H_2_O; (**d**) CaCl_2_-KNO_3_/H_2_O; (**e**) CaCl_2_-LiNO_3_/H_2_O; and, (**f**) CaCl_2_-LiNO_3_-KNO_3_/H_2_O.

The *T_C_* of CaCl_2_-NaCl/H_2_O, CaCl_2_-KCl/H_2_O, CaCl_2_-LiCl/H_2_O, CaCl_2_-KNO_3_/H_2_O, CaCl_2_-LiNO_3_/H_2_O, and CaCl_2_-LiNO_3_-KNO_3_/H_2_O were measured. Figure 3 gives the comparison of *T_C_* between these CaCl_2_-based working pairs and CaCl_2_/H_2_O. Here, the concentration is the solution’s total solute mass concentration.

Figure 3a shows CaCl_2_-NaCl/H_2_O with adding 5.0 g NaCl to CaCl_2_/H_2_O, in which CaCl_2_ were from 42.9 g to 61.3 g and H_2_O was 95.0 g. The crystallization temperatures of CaCl_2_-NaCl/H_2_O were higher than those of CaCl_2_/H_2_O under the same concentrations.

Figure 3b shows CaCl_2_-KCl/H_2_O with adding 5.0 g KCl to CaCl_2_/H_2_O, in which CaCl_2_ were from 78.6 g to 117.4 g and H_2_O was 95.0 g. The crystallization temperatures of CaCl_2_-KCl/H_2_O were approximately 20.0 °C lower than those of CaCl_2_/H_2_O under the same concentrations.

Figure 3c shows CaCl_2_-LiCl/H_2_O with adding 10.0 g LiCl to CaCl_2_/H_2_O, in which CaCl_2_ were from 66.7 g to 100.0 g and H_2_O was 90.0 g. The crystallization temperature of CaCl_2_-LiCl/H_2_O was reduced greatly when compared with that of CaCl_2_/H_2_O at 50.0 wt.%, whereas with the concentration increasing, the effect of LiCl addition on the crystallization temperature obviously decreased.

Figure 3d shows CaCl_2_-KNO_3_/H_2_O with adding 10.0 g KNO_3_ to CaCl_2_/H_2_O, in which CaCl_2_ were from 78.6 g to 117.4 g and H_2_O was 90.0 g. The crystallization temperatures of CaCl_2_-KNO_3_/H_2_O were lower than those of CaCl_2_/H_2_O under the same concentrations. Moreover, it decreased with increasing concentration in the range of 49.0 wt.% to 53.5 wt.%, whereas it increased with further increasing concentration.

Figure 3e shows CaCl_2_-LiNO_3_/H_2_O with adding 35.0 g LiNO_3_ to CaCl_2_/H_2_O, in which CaCl_2_ were from 42.9 g to 100.0 g and H_2_O was 65.0 g. The crystallization temperatures of CaCl_2_-LiNO_3_/H_2_O were significantly reduced when compared with those of CaCl_2_/H_2_O under the same concentrations. Corresponding to the concentrations ranging from 55.0 wt.% to 62.0 wt.%, which is a practical concentration range for an absorption refrigeration cycle, the crystallization temperatures of CaCl_2_-LiNO_3_/H_2_O were from -10.0 °C to 7.0 °C, which are sufficiently low to solve the absorbent crystallization problem in summer. However, the addition amount of 35.0 g LiNO_3_ was relatively large, and it is a disadvantage from the aspect of cost due to LiNO_3_ being much more expensive than CaCl_2_.

To depress the cost increase, a part of LiNO_3_ was replaced with KNO_3_ for CaCl_2_-LiNO_3_/H_2_O. Figure 3f shows CaCl_2_-LiNO_3_-KNO_3_/H_2_O with adding 25.0 g LiNO_3_ and 5.0 g KNO_3_ to CaCl_2_/H_2_O, in which CaCl_2_ were from 66.7 g to 117.4 g and H_2_O was 70.0 g. A reduction of crystallization temperature up to 30.0 °C was achieved from 58.0 wt.% to 65.0 wt.%, which indicated that the crystallization problem would not occur in this concentration range.

### 3.3. Measurement of Saturated Vapor Pressure p

*p* of CaCl_2_-LiNO_3_/H_2_O and CaCl_2_-LiNO_3_-KNO_3_/H_2_O with different mass ratios were measured and are shown in Table 4 and Table 5.

The measured *p* was fitted by Equation (1) [33,34,35].

(1) $$\log p=\overset{4}{\underset{i=0}{\sum }}\left[A_{i}+B_{i}/(T+273.15-C_{i})\right]w^{i}$$

where *A_i_*, *B_i_*, and *C_i_* are the regression parameters. Equation (2) obtains the average absolute relative deviation (AARD) between the measured values and the fitted values.

(2) $$\mathrm{AARD}=1/N\sum_{i=1}^{N}\left|\left(P_{\exp}-P_{fit}\right)/P_{fit}\right|$$

where *N* is total number of data, *P*_exp_ is the measured or obtained value, and *P_fit_* is the fitted value.

The regression parameters and AARD were obtained and are shown in Table 6 and Table 7, respectively.

**Figure 4:**
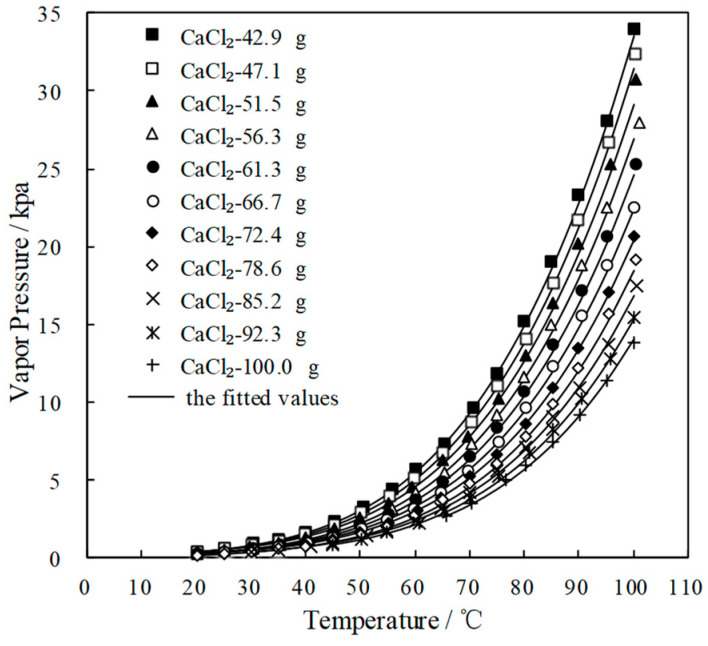
*p* of CaCl_2_-LiNO_3_/H_2_O with different mass ratio.

**Figure 5:**
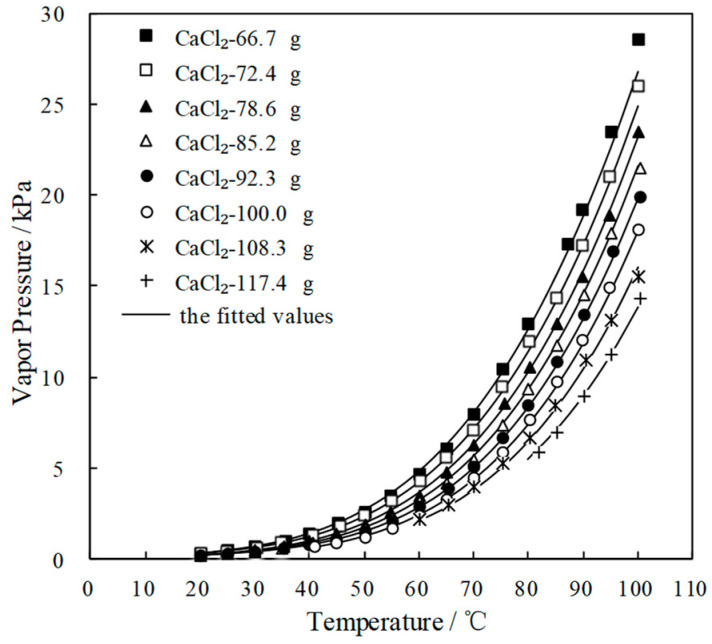
*p* of CaCl_2_-LiNO_3_-KNO_3_/H_2_O with different mass ratio.

Figure 4 and Figure 5 plot the measured *p* and fitted value of CaCl_2_-LiNO_3_/H_2_O and CaCl_2_-LiNO_3_-KNO_3_/H_2_O, respectively. The fitted value agreed well with the measured *p*. 60.2 wt.% for CaCl_2_(63.2 g)-LiNO_3_(35.0 g)/H_2_O(65.0 g) and 60.5 wt.% for CaCl_2_(77.3 g)-LiNO_3_(25.0 g)-KNO_3_(5.0 g)/H_2_O(70.0 g) were obtained, respectively, by Equation (1) at 37.0 °C and 0.872 kPa, which are the typical absorption temperature and absorption pressure for a refrigeration cycle. Meanwhile, the absorption temperatures of these two working pairs were 70.5 °C and 69.2 °C, respectively, at 6.290 kPa, which is the typical generation pressure. Therefore, CaCl_2_(77.3 g)-LiNO_3_(25.0 g)-KNO_3_(5.0 g)/H_2_O(70.0 g), with a solute mass ratio of 15.5:5:1, had been proposed as an alternative working pair for LiBr/H_2_O. The proposed working pair is expressed as CaCl_2_-LiNO_3_-KNO_3_(15.5:5:1)/H_2_O in this paper.

The *p* of this working pair was measured in order to analyze the cycle performance with CaCl_2_-LiNO_3_-KNO_3_(15.5:5:1)/H_2_O, as shown in Table 8.

The measured *p* was fitted by Equation (3) and the AARD was obtained to be 1.82% by Equation (2).

(3) $$\begin{array}{l} \log p=1.243-8.293/(T+273.15+3.466\times 10^{3})+\\ (1.698\times 10^{-1}-3.894\times 10/(T+273.15+1.944\times 10^{2}))\times w+\\ (-1.503\times 10^{-3}+3.797\times 10^{-1}/(T+273.15+2.836\times 10^{2}))\times w^{2} \end{array}$$

**Figure 6:**
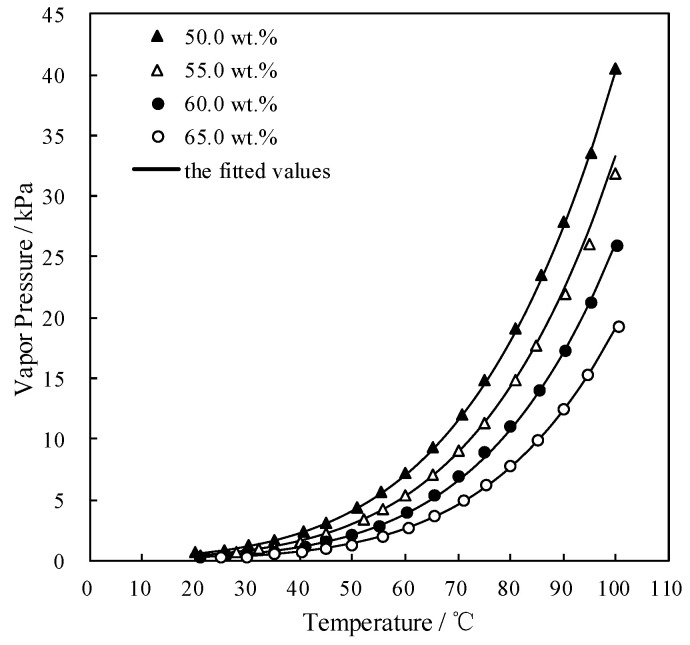
*p* of CaCl_2_-LiNO_3_-KNO_3_(15.5:5:1)/H_2_O.

Figure 6 shows the measured *p* and fitted value. The measured *p* agreed well with the fitted value, which indicated that the *p* of CaCl_2_-LiNO_3_-KNO_3_(15.5:5:1)/H_2_O could be obtained with the given corresponding concentration and temperature.

**Figure 7:**
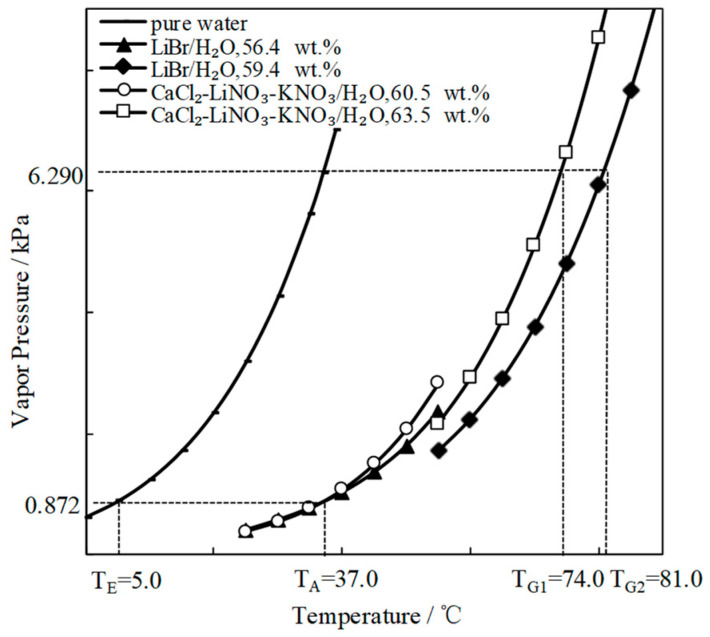
Comparison of the refrigeration characteristic between LiBr/H_2_O and CaCl_2_-LiNO_3_-KNO_3_(15.5:5:1)/H_2_O.

Figure 7 compares the refrigeration characteristic of CaCl_2_-LiNO_3_-KNO_3_(15.5:5:1)/H_2_O and LiBr/H_2_O. The generation temperature of CaCl_2_-LiNO_3_-KNO_3_(15.5:5:1)/H_2_O was 74.0 °C, which was 7.0 °C lower than that of LiBr/H_2_O. In other words, the temperature that is required for the driving heat source could be reduced by 7.0 °C through using CaCl_2_-LiNO_3_-KNO_3_(15.5:5:1)/H_2_O instead of LiBr/H_2_O.

### 3.4. Measurement of Density ρ

*ρ* of CaCl_2_-LiNO_3_-KNO_3_(15.5:5:1) /H_2_O was measured by a capillary pycnometer method. Table 9 lists the results.

The measured *ρ* of CaCl_2_-LiNO_3_-KNO_3_(15.5:5:1)/H_2_O was fitted by Equation (4) and AARD was obtained to be 0.22% by Equation (2).

(4) $$\begin{array}{l} \rho =1.923\times 10^{2}-1.139\times (T+273.15)+1.690\times 10^{-3}\times {\left(T+273.15\right)}^{2}\\ +(-1.034\times 10^{3}+6.162\times (T+273.15)-9.146\times 10^{-3}\times {\left(T+273.15\right)}^{2})\times w\\ +(1.859\times 10^{3}-1.107\times 10\times (T+273.15)+1.642\times 10^{-2}\times {\left(T+273.15\right)}^{2})\times w^{2}\\ +(-1.108\times 10^{3}+6.595\times (T+273.15)-9.781\times 10^{-3}\times {\left(T+273.15\right)}^{2})\times w^{3} \end{array}$$

**Figure 8:**
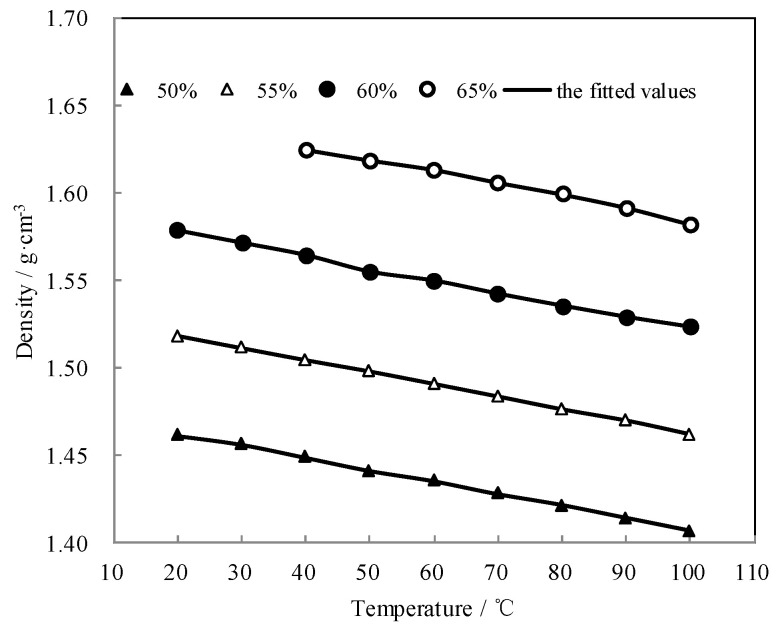
*ρ* of CaCl_2_-LiNO_3_-KNO_3_(15.5:5:1)/H_2_O.

Figure 8 shows the measured *ρ* and the fitted value. The measured *ρ* was highly consistent with the fitted value. The density linearly decreased with the temperature increasing, and it increased with the concentration increasing.

### 3.5. Measurement of Viscosity η

*η* of CaCl_2_-LiNO_3_-KNO_3_(15.5:5:1)/H_2_O was measured by the Ubbelohde capillary viscometer method. Table 10 shows the results.

The measured *η* of CaCl_2_-LiNO_3_-KNO_3_(15.5:5:1)/H_2_O was fitted by Equation (5) and the AARD was obtained to be 0.82% by Equation (2).

(5) $$\begin{array}{l} \eta =8.099\times 10-4.721\times 10^{4}/(T+273.15)+8.480\times 10^{6}/{\left(T+273.15\right)}^{2}\\ +(-2.286\times 10^{2}+1.169\times 10^{5}/(T+273.15)-2.278\times 10^{7}/{\left(T+273.15\right)}^{2})\times w\\ +(2.534\times 10-1.816\times 10^{4}/(T+273.15)-4.422\times 10^{5}/{\left(T+273.15\right)}^{2})\times w^{2}\\ +(2.224\times 10^{2}+4.127\times 10^{5}/(T+273.15)+2.716\times 10^{7}/{\left(T+273.15\right)}^{2})\times w^{3} \end{array}$$

**Figure 9:**
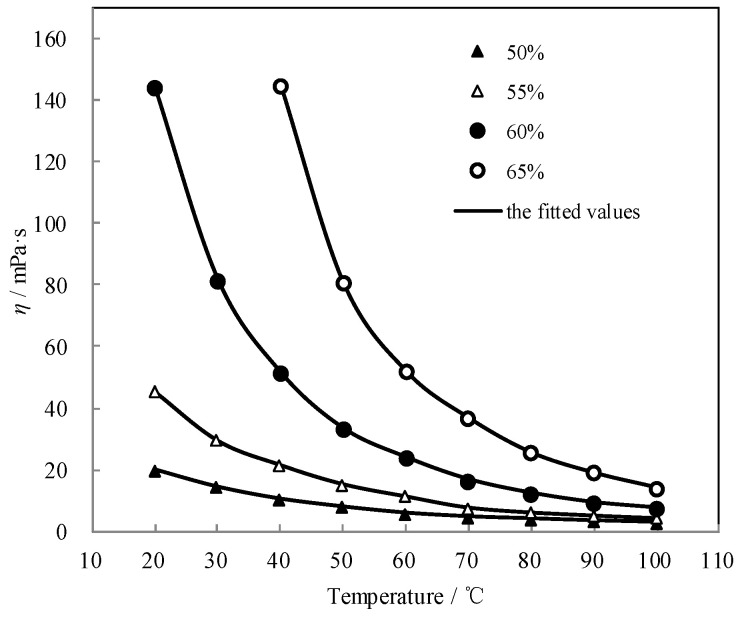
*η* of CaCl_2_-LiNO_3_-KNO_3_(15.5:5:1)/H_2_O.

Figure 9 shows the measured *η* and the fitted value. The measured *η* agreed well with the fitted value. *η* exponentially decreased with the temperature increasing, whereas it increased with the concentration increasing.

### 3.6. Measurement of Specific Heat Capacity C_p_

*C_p_* of CaCl_2_-LiNO_3_-KNO_3_(15.5:5:1)/H_2_O was measured with a micro reaction calorimeter. Table 11 lists the results.

The measured *C_p_* was fitted by Equation (6) and AARD was obtained to be 0.21% by Equation (2).

(6) $$\begin{array}{l} C_{p}=2.575+1.951\times 10^{-2}\times (T+273.15)-2.200\times 10^{-4}\times {\left(T+273.15\right)}^{2}\\ +\left(1.121\times 10^{-2}-6.433\times 10^{-4}\times (T+273.15)+7.593\times 10^{-6}\times {\left(T+273.15\right)}^{2}\right)w\\ +(-3.259\times 10^{-4}+5.570\times 10^{-6}\times (T+273.15)-7.020\times 10^{-8}\times {\left(T+273.15\right)}^{2})w^{2} \end{array}$$

**Figure 10:**
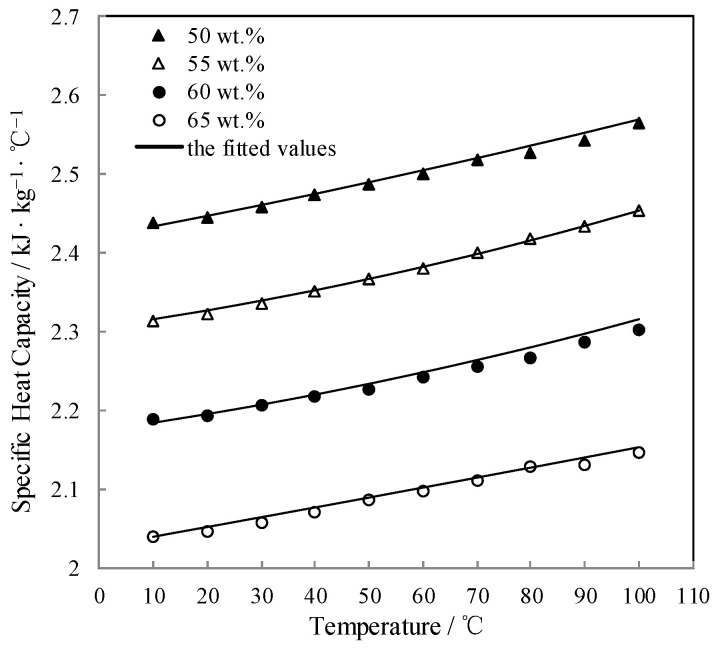
*C_p_* of CaCl_2_-LiNO_3_-KNO_3_(15.5:5:1) /H_2_O.

Figure 10 shows the measured *C_p_* and the fitted value. *C_p_* linearly increased with increasing the temperature.

### 3.7. Calculation of Specific Enthalpy h

#### 3.7.1. C_p_ of CaCl_2_, LiNO_3_, KNO_3_ and H_2_O

The *C_p_* of solid KNO_3_ was measured and is shown in Table 12, *C_p_* of CaCl_2_, LiNO_3_, and H_2_O are given in Reference Literature [29].

#### 3.7.2. Measurement of Dissolution Enthalpy Δ*H_mix_*

Δ*H*_mix_ of KNO_3_, LiNO_3_, and CaCl_2_ with a mass ratio of 15.5:5:1 were measured at 25.0 °C and are shown in Table 13.

#### 3.7.3. Calculation of Specific Enthalpy *h*

*h* of CaCl_2_-LiNO_3_-KNO_3_(15.5:5:1) /H_2_O can be obtained from the measured *C_p_* and Δ*H*_mix_ [25,36,37]. Table 14 lists the obtained results.

The obtained *h* was fitted by Equation (7) and AARD was obtained to be 0.07% by Equation (2).

(7) $$\begin{array}{l} h=3.151\times 10^{2}-1.193\times w+6.998\times 10^{-3}\times w^{2}\\ +(2.971-2.553\times 10^{-3}\times w-1.817\times 10^{-4}\times w^{2})(T+273.15)\\ +(-1.775\times 10^{-3}+1.198\times 10^{-4}\times w-1.304\times 10^{-6}\times w^{2}){\left(T+273.15\right)}^{2} \end{array}$$

**Figure 11:**
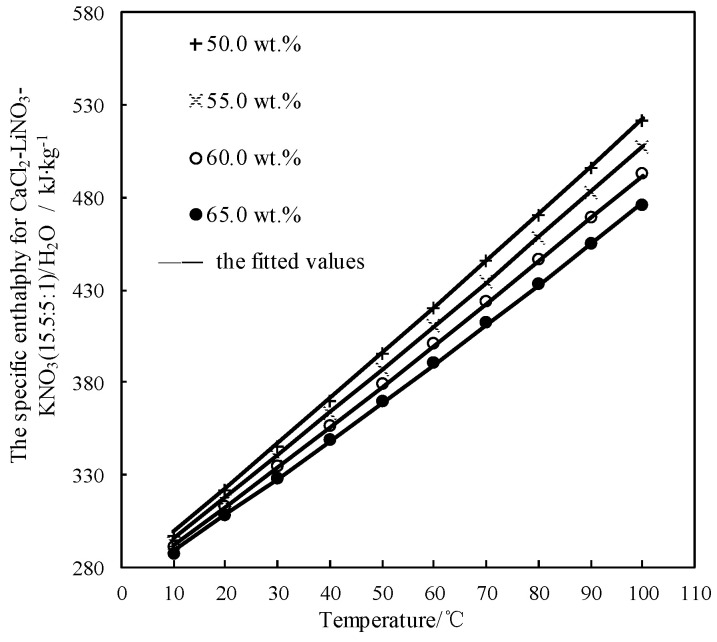
*h* of CaCl_2_-LiNO_3_-KNO_3_(15.5:5:1) /H_2_O.

Figure 11 shows the obtained *h* and the fitted value. *h* linearly increased with increasing the temperature, and the slope of line slightly increased with reducing the concentration.

### 3.8. Calculation of Specific Entropy s

*s* of a solution can be also obtained from the measured *C_p_* and Δ*H*_mix_ [38]. *s* of CaCl_2_-LiNO_3_-KNO_3_(15.5:5:1) /H_2_O was obtained and is shown in Table 15.

The obtained *s* of CaCl_2_-LiNO_3_-KNO_3_(15.5:5:1) /H_2_O was fitted by Equation (8) and the AARD was obtained to be 0.84% by Equation (2).

(8) $$\begin{array}{l} s=-2.662\times 10+1.370\times 10^{2}\times w-2.828\times 10^{2}\times w^{2}+1.967\times 10^{2}\times w^{3}\\ +(2.333\times 10^{-1}+1.315\times w+2.735\times w^{2}-1.892\times w^{3})(T+273.15)+\\ (-6.999\times 10^{-4}+4.199\times 10^{-3}\times w-8.788\times 10^{-3}\times w^{2}+6.062\times 10^{-3}\times w^{3}){\left(T+273.15\right)}^{2}\\ +(7.282\times 10^{-7}-4.461\times 10^{-6}\times w+9.373\times 10^{-6}\times w^{2}-6.469\times 10^{-6}\times w^{3}){\left(T+273.15\right)}^{3} \end{array}$$

**Figure 12:**
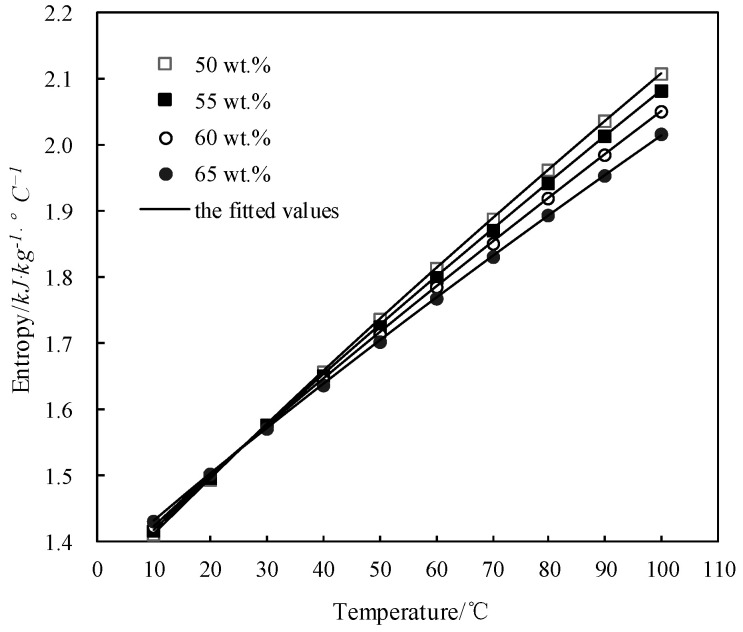
*s* of CaCl_2_-LiNO_3_-KNO_3_(15.5:5:1)/H_2_O.

Figure 12 shows the obtained *s* and the fitted value. *s* increased with the temperature increasing and decreased with the concentration increasing when the temperature was above 28 °C, whereas it changed little with the concentration when the temperature was below 28 °C.

### 3.9. Application for an Absorption Refrigeration Cycle

#### 3.9.1. Absorption Refrigeration Cycle Using CaCl_2_-LiNO_3_-KNO_3_(15.5:5:1)/H_2_O

**Figure 13:**
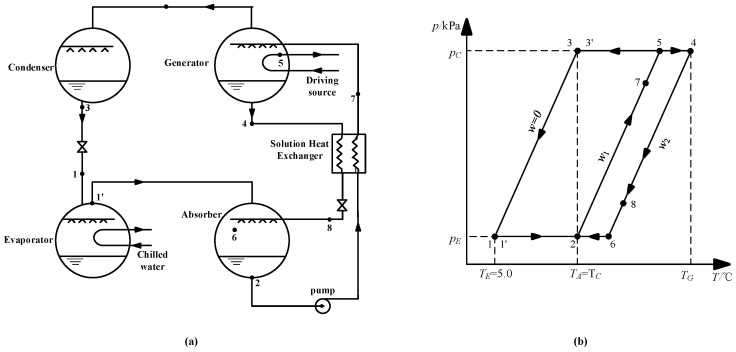
(**a**) Schematic of the absorption refrigeration cycle; (**b**) *P-T* diagram of the absorption refrigeration cycle.

Figure 13a shows the schematic of an absorption refrigeration cycle. Figure 13b is the *P-T* diagram of the cycle, and the points that are marked in the two diagrams are one-to-one correspondence.

The working conditions are given, as follows: the evaporation temperature was 5.0 °C; the absorption temperature and condensation temperature were 37.0 °C; and, the evaporation and condensation pressures were 0.872 kPa and 6.290 kPa, respectively. The concentration of dilute solution for CaCl_2_-LiNO_3_-KNO_3_(15.5:5:1)/H_2_O was figured out to be 60.5 wt.% by Equation (3), and the strong solution was 63.5 wt.%, with a concentration difference of 3.0 wt.%, thus, the generation temperature of the cycle was determined to be 74.0 °C by Equation (3). The same method was applied to calculate the generation temperature while using LiBr/H_2_O and other CaCl_2_-based working pairs, including CaCl_2_-LiBr-LiNO_3_-KNO_3_(16.2:2:2:1)/H_2_O, CaCl_2_-LiNO_3_-LiBr(8.72:1:1)/H_2_O, and CaCl_2_-LiBr(1.35:1)/H_2_O. Table 16 lists the results.

As seen in Table 16, the generation temperature was reduced by 7.0 °C through the use of CaCl_2_-LiNO_3_-KNO_3_(15.5:5:1)/H_2_O instead of LiBr/H_2_O. The generation temperature differences between the four CaCl_2_-based working pairs were relatively small.

#### 3.9.2. Analysis of COP and Exergy Efficiency

To analyze the performance of a refrigeration cycle with CaCl_2_-LiNO_3_-KNO_3_(15.5:5:1)/H_2_O, the state parameters of typical points in Figure 13 were obtained by Equations (3), (7) and (8). Table 17 lists the results.

The coefficient of performance (COP) for the absorption refrigeration cycle can be defined as:

(9) $$\mathrm{COP}=\frac{Q_{E}}{Q_{G}}=\frac{h_{1{}^{\prime }}-h_{3}}{h_{4{}^{\prime }}-h_{4}+\alpha \left(h_{4}-h_{7}\right)}$$

where *α* represents circulating ratio.

COP was obtained to be 0.801 when using CaCl_2_-LiNO_3_-KNO_3_(15.5:5:1)/H_2_O as the working pair. The COPs for other working pairs were obtained with the same method, and the results are listed in Table 18.

Table 18 shows that, through using CaCl_2_-LiNO_3_-KNO_3_(15.5:5:1)/H_2_O instead of LiBr/H_2_O, the COP was improved by 0.04. Moreover, the exergy destruction in each part of the cycle were analyzed to further compare the performance between CaCl_2_-LiNO_3_-KNO_3_(15.5:5:1)/H_2_O and LiBr/H_2_O. Exergy is defined as the maximum possible reversible work that can be obtained from a stream:

(10) $$E=(h-h_{0})-(T_{0}+273.15)(s-s_{0})$$

where *T*_0_ represents the environment temperature that was taken as 25 °C in this paper.

The exergy destructions for each part of the absorption refrigeration cycle were obtained as follows [39].

Evaporator:

(11) $$\Delta E_{E}{=D}_{3}E_{3}-D_{1{}^{\prime }}E_{1{}^{\prime }}+Q_{E}(\frac{T_{0}}{T_{E}}-1)$$

Condenser:

(12) $$\Delta E_{C}{=D}_{4{}^{\prime }}E_{4{}^{\prime }}-D_{3}E_{3}-Q_{C}(1-\frac{T_{0}}{T_{C}})$$

Absorber:

(13) $$\Delta E_{A}{=D}_{8}E_{8}+D_{1{}^{\prime }}E_{1{}^{\prime }}-D_{2}E_{2}-Q_{A}(1-\frac{T_{0}}{T_{A}})$$

Generator:

(14) $$\Delta E_{G}{=D}_{7}E_{7}-D_{4}E_{4}-D_{4{}^{\prime }}E_{4{}^{\prime }}+Q_{G}(1-\frac{T_{0}}{T_{G}})$$

Heat exchanger:

(15) $$\Delta E_{HEX}{=D}_{2}E_{2}+D_{4}E_{4}-D_{7}E_{7}-D_{8}E_{8}$$

Table 19 compares the exergy destructions of the absorption cycle with CaCl_2_-LiNO_3_-KNO_3_(15.5:5:1)/H_2_O and LiBr/H_2_O. Except the exergy destruction of the evaporator was equal because of the same evaporation condition, the exergy destructions of other parts for CaCl_2_-LiNO_3_-KNO_3_(15.5:5:1)/H_2_O were lower than those for LiBr/H_2_O. For the absorption refrigeration cycle, the exergy efficiency (*η_E_*) can be defined as:

(16) $$\eta_{E}=\frac{Q_{E}(T_{E}/T_{0}-1)}{Q_{G}(1-T_{Q}/T_{0})}$$

The *η_E_* of the absorption refrigeration cycle with CaCl_2_-LiNO_3_-KNO_3_(15.5:5:1)/H_2_O and LiBr/H_2_O were obtained to be 0.327 and 0.272, respectively. When compared with COP, the difference in exergy efficiency between the two working pairs was more distinct, which further showed the advantage of CaCl_2_-LiNO_3_-KNO_3_(15.5:5:1)/H_2_O as an alternative working pair.

**Figure 14:**
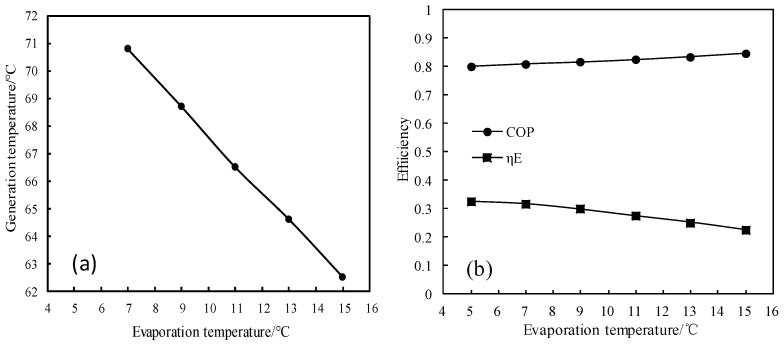
(**a**) Variation of the generation temperature with the evaporation temperature; and, (**b**) Variations of COP and *η_E_* with the evaporation temperature.

**Figure 15:**
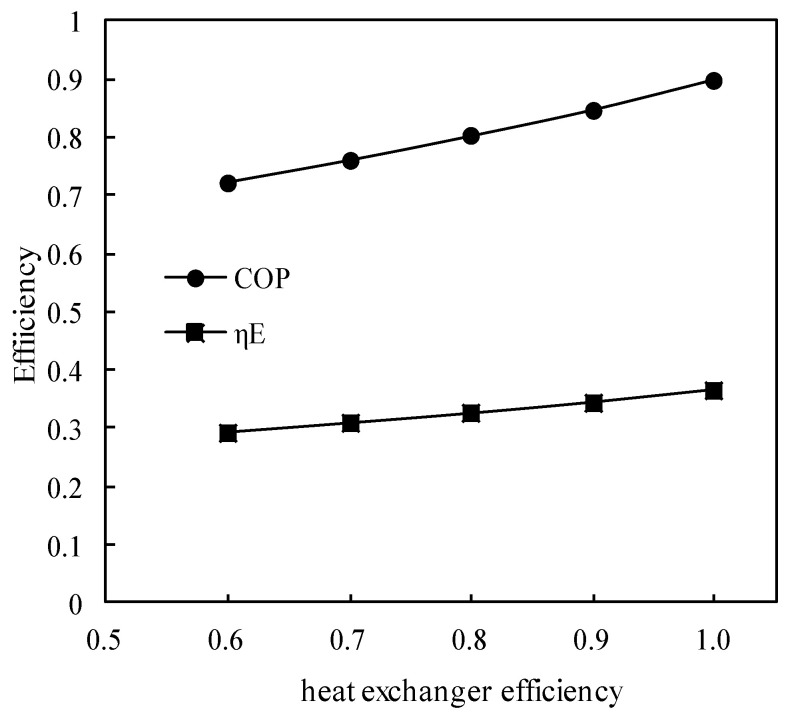
Variations of COP and *η_E_* with the heat exchanger efficiency.

Figure 14 and Figure 15 show the changes of generation temperature and efficiencies (COP and *η_E_*) for CaCl_2_-LiNO_3_-KNO_3_(15.5:5:1)/H_2_O, with the evaporation temperature varying from 5 °C to 15 °C. As shown in Figure 14a, the generation temperature decreased almost linearly with increasing the evaporation temperature. As shown in Figure 14b, the COP of the absorption refrigeration cycle increased with the evaporation temperature increasing, whereas the exergy efficiency decreased with the evaporation temperature increasing.

Figure 15 shows the variations of COP and *η_E_* with the solution heat exchanger efficiency. COP and *η_E_* increased almost linearly with the heat exchanger efficiency increasing, and the increasing slope of COP was greater than that of *η_E_*.

### 3.10. Measurement of Corrosion Rate R_C_

**Figure 16:**
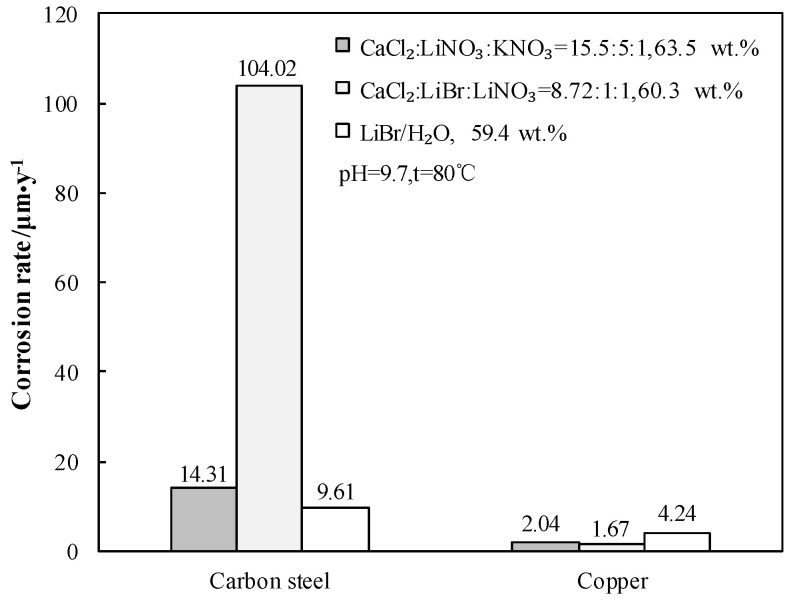
*R_C_* of carbon steel and copper for CaCl_2_-LiNO_3_-KNO_3_(15.5:5:1)/H_2_O, CaCl_2_-LiNO_3_-LiBr(8.72:1:1)/H_2_O and LiBr/H_2_O.

Generally, carbon steel is used as the structural material and copper is used as the heat exchange material for absorption heat pump. The *R_C_* of carbon steel and copper in 63.5 wt.% solution of CaCl_2_-LiNO_3_-KNO_3_(15.5:5:1)/H_2_O were measured at 80.0 °C and pH 9.7. Figure 16 gives the comparison of *R_C_* in 63.5 wt.% solution of CaCl_2_-LiNO_3_-KNO_3_(15.5:5:1)/H_2_O, 59.4 wt.% solution of LiBr/H_2_O, and 60.3 wt.% solution of CaCl_2_-LiNO_3_-LiBr(8.72:1:1)/H_2_O.

Figure 16 shows that the *R_c_* of carbon steel in 63.5 wt.% solution of CaCl_2_-LiNO_3_-KNO_3_(15.5:5:1)/H_2_O was 14.31 μm∙y^−1^. Although the corrosivity of CaCl_2_-LiNO_3_-KNO_3_(15.5:5:1)/H_2_O to carbon steel was stronger than that of LiBr/H_2_O, it was still acceptable for practical applications. On the other hand, the corrosivity of CaCl_2_-LiNO_3_-LiBr(8.72:1:1)/H_2_O to carbon steel was too strong to be applied, even though it had the lowest generation temperature among the CaCl_2_-based working pairs. The *R_c_* of copper in 63.5 wt.% solution of CaCl_2_-LiNO_3_-KNO_3_(15.5:5:1)/H_2_O was 2.04 μm∙y^−1^, which was smaller than that in 59.4 wt.% solution of LiBr/H_2_O and it could meet the requirements for engineering applications.

## 4. Conclusions

1. When compared with LiBr/H_2_O, for an identical adsorption temperature at 0.872 kPa, which is a typical pressure of absorber, CaCl_2_/H_2_O had a lower absorption temperature at 6.290 kPa, which is a typical pressure of generator, meaning that CaCl_2_/H_2_O basically had a better refrigeration characteristic for an absorption refrigeration cycle. However, the absorption ability of CaCl_2_/H_2_O was not strong enough for achieving an evaporation temperature of 5 °C or lower, because of its high crystallization temperature.
2. The crystallization temperature was significantly lowered when combining CaCl_2_/H_2_O with LiNO_3_ or LiNO_3_+KNO_3_. As a result, the absorption ability of CaCl_2_-LiNO_3_/H_2_O or CaCl_2_-LiNO_3_-KNO_3_/H_2_O was essentially improved.
3. For an absorption refrigeration cycle using CaCl_2_-LiNO_3_-KNO_3_(15.5:5:1)/H_2_O as the working pair, the generation temperature that is required for achieving an evaporation temperature of 5 °C was 74.0 °C, which was 7.0 °C lower than that using LiBr/H_2_O.
4. When compared with LiBr/H_2_O under the same conditions, COP and *η_E_* of the absorption refrigeration cycle with CaCl_2_-LiNO_3_-KNO_3_(15.5:5:1)/H_2_O were improved by 0.04 and 0.06, respectively.
5. *R_C_* of carbon steel and copper in 63.5 wt.% solution of CaCl_2_-LiNO_3_-KNO_3_(15.5:5:1)/H_2_O at 80.0 °C and pH 9.7 were 14.31 and 2.04 μm∙y^−1^, respectively, which indicated that the corrosivity of the proposed working pair could meet the requirements for practical applications.

## Figures and Tables

**Table 1:** Purity of the used regents.

Reagent	Mass Concentration Purity	Provenance
CaCl_2_	>0.96	Sinopharm Chemical Reagent Beijing
NaCl	>0.995	Sinopharm Chemical Reagent Beijing
KNO_3_	>0.99	Sinopharm Chemical Reagent Beijing
KCl	>0.995	Sinopharm Chemical Reagent Beijing
LiCl	>0.95	Tianjin Jinke Chemical
LiNO_3_	>0.995	Tianjin Jinke Chemical
Ultrapure water		Home-made

**Table 2:** Chemical compositions of carbon steel and copper.

Component	*C*	*Mn*	*Si*	*P*	*S*	*Zn*	*Pb*	*Sn*	*Fe*	*Cu*
Carbon steel Q235	0.16	0.53	0.3	0.035	0.04	--	--	--	balance	--
Copper T6	--	--	0.006	--	0.01	0.005	0.05	0.05	0.05	balance

**Table 3:** Measuring range and accuracy of main instruments.

Instrument	Parameter	Accuracy
Analytical balance	0–2100 g	±0.1 g
Precision thermostat	−30–150 °C	±0.5 °C
Oil bath	20–300 °C	±1.0 °C
Digital absolute pressure gauge	0–110 kPa	±0.01 kPa
Precision viscometer oil bath	0–230 °C	±0.05 °C
Capillary pycnometer	50 mL	±0.03%
Ubbelohde capillary viscometer	0.36 mm, 0.46 mm, 0.58 mm, 073 mm	±0.02%
Micro reaction calorimeter	0–180 °C	±0.001 °C

**Table 4:** *p* of CaCl_2_-LiNO_3_/H_2_O with different mass ratio.

*x*/g		Saturated Vapor Pressure *p* (kPa) at Each Temperature *T* (°C) CaCl_2_(*x*)-LiNO_3_(35.0 g)/H_2_O(65.0 g)								
42.9	*T*	20.0	25.0	30.5	35.0	40.0	45.3	50.4	55.7	60.2
	*p*	0.457	0.641	0.934	1.256	1.693	2.369	3.278	4.431	5.707
	*T*	65.3	70.8	75.0	80.0	85.0	90.0	95.0	100.0	
	*p*	7.376	9.632	11.825	15.204	19.002	23.331	28.101	34.022	
47.1	*T*	20.0	25.0	30.2	35.0	40.1	45.3	50.2	55.5	59.9
	*p*	0.419	0.585	0.834	1.138	1.535	2.139	2.948	3.998	5.136
	*T*	65.2	70.3	75.1	80.2	85.2	90.0	95.4	100.3	
	*p*	6.823	8.739	11.035	14.118	17.692	21.770	26.701	32.375	
51.5	*T*	20.0	25.0	29.9	35.0	40.1	45.2	50.0	55.2	59.6
	*p*	0.372	0.520	0.733	1.010	1.377	1.910	2.617	3.564	4.565
	*T*	65.1	69.8	75.2	80.4	85.3	90.0	95.8	100.6	
	*p*	6.269	7.846	10.244	13.031	16.382	20.208	25.300	30.728	
56.3	*T*	20.0	25.0	30.3	35.3	40.1	45.1	50.0	55.7	60.1
	*p*	0.330	0.460	0.674	0.921	1.233	1.688	2.336	3.217	4.188
	*T*	65.3	70.5	75.0	79.9	85.0	90.4	95.0	101.0	
	*p*	5.523	7.333	9.228	11.688	14.998	18.869	22.500	27.967	
61.3	*T*	20.1	25.0	30.3	35.2	40.2	45.1	50.1	55.3	60.0
	*p*	0.295	0.419	0.603	0.826	1.124	1.531	2.113	2.844	3.704
	*T*	65.0	70.2	75.1	80.1	85.1	90.5	95.1	100.5	
	*p*	4.894	6.490	8.349	10.671	13.678	17.233	20.685	25.265	
66.7	*T*	20.2	25.0	30.3	35.1	40.2	45.1	50.1	54.9	59.9
		0.269	0.378	0.552	0.752	1.024	1.375	1.860	2.471	3.220
	*T*	64.6	69.8	75.2	80.2	85.2	90.5	95.2	100.0	
		4.265	5.647	7.470	9.654	12.358	15.597	18.870	22.562	
72.4	*T*	20.1	25.0	30.1	35.0	40.0	44.9	50.0	55.2	60.4
	*p*	0.247	0.341	0.492	0.671	0.899	1.186	1.657	2.237	3.092
	*T*	64.6	70.0	75.0	80.4	85.1	90.0	95.5	100.2	
	*p*	3.930	5.268	6.678	8.678	10.960	13.507	17.127	20.709	
78.6	*T*	20.1	25.0	30.0	35.1	40.0	45.1	50.1	55.0	59.9
	*p*	0.218	0.298	0.430	0.592	0.804	1.106	1.515	2.019	2.722
	*T*	65.2	70.2	74.9	80.2	85.1	90.0	95.5	100.3	
	*p*	3.709	4.810	6.099	7.805	9.901	12.206	15.709	19.204	
85.2	*T*	30.1	35.0	41.1	45.0	51.1	54.9	60.0	64.9	69.9
	*p*	0.390	0.529	0.768	0.981	1.420	1.780	2.385	3.205	4.185
	*T*	75.0	80.2	85.3	90.2	95.4	100.6			
	*p*	5.624	7.135	9.098	10.996	13.756	17.535			
92.3	*T*	45.1	50.1	54.9	60.8	65.4	70.2	75.5	81.1	85.1
	*p*	0.886	1.188	1.645	2.303	3.048	3.903	5.226	6.777	8.311
	*T*	90.5	95.8	100.0						
	*p*	10.276	12.820	15.447						
100.0	*T*	65.7	70.4	76.7	80.2	85.1	90.2	95.2	100.1	
	*p*	2.711	3.552	5.050	6.000	7.432	9.260	11.383	13.858	

**Table 5:** *p* of CaCl_2_-LiNO_3_-KNO_3_/H_2_O with different mass ratio.

*y*/g		Saturated Vapor Pressure *p* (kPa) at Each Temperature *T* (°C) CaCl_2_(*y*)-LiNO_3_(25.0 g)-KNO_3_(5.0 g)/H_2_O(70.0 g)								
66.7	*T*	20.0	25.0	30.1	35.5	40.0	45.3	50.0	54.9	60.0
	*p*	0.352	0.511	0.741	1.064	1.434	1.987	2.638	3.508	4.660
	*T*	65.1	70.1	75.2	80.0	87.2	89.9	95.0	100.0	
	*p*	6.119	8.024	10.490	13.001	17.346	19.223	23.450	28.590	
72.4	*T*	20.0	25.0	30.2	34.9	40.6	45.6	50.0	55.2	60.4
	*p*	0.315	0.455	0.650	0.911	1.353	1.850	2.403	3.250	4.283
	*T*	65.1	69.9	75.2	80.3	85.0	89.7	94.9	100.0	
	*p*	5.560	7.106	9.469	11.911	14.327	17.200	21.021	26.025	
78.6	*T*	20.0	25.0	29.9	35.1	40.0	45.0	50.0	54.9	60.0
	*p*	0.259	0.371	0.530	0.762	1.049	1.427	1.940	2.634	3.552
	*T*	65.1	69.8	75.5	80.1	85.0	89.9	94.9	100.1	
	*p*	4.825	6.306	8.546	10.555	12.913	15.540	18.898	23.535	
85.2	*T*	20.0	25.0	30.0	35.0	40.5	45.0	50.0	55.0	60.2
	*p*	0.219	0.312	0.445	0.627	0.928	1.239	1.740	2.311	3.200
	*T*	65.0	69.8	75.1	80.0	85.1	90.0	95.2	100.2	
	*p*	4.205	5.499	7.402	9.378	11.789	17.909	21.505		
92.3	*T*	20.0	25.0	30.0	35.4	39.9	45.0	50.0	55.1	60.1
	*p*	0.198	0.285	0.399	0.581	0.793	1.136	1.563	2.150	2.925
	*T*	65.2	70.0	75.3	79.9	85.2	90.0	95.5	100.2	
	*p*	3.875	5.048	6.683	8.448	10.859	13.429	16.906	19.867	
100.0	*T*	41.0	44.9	50.0	55.0	60.2	65.0	70.1	75.1	80.3
	*p*	0.673	0.889	1.244	1.730	2.454	3.349	4.519	5.911	7.656
	*T*	85.2	89.9	94.9	99.9					
	*p*	9.761	12.060	14.960	18.163					
108.3	*T*	60.0	65.2	70.0	75.2	80.1	84.8	90.4	95.1	100.1
	*p*	2.205	3.045	4.051	5.252	6.686	8.464	10.993	13.110	15.587
117.4	*T*	82.0	85.1	90.0	95.0	100.3				
	*p*	5.921	6.971	9.009	11.303	14.387				

**Table 6:** Regression parameters for CaCl_2_-LiNO_3_/H_2_O and average absolute relative deviation (AARD) value.

*i*	*A_i_*	*B_i_*	*C_i_*	AARD
0	4.896 × 10^−1^	−1.985 × 10^0^	−3.624 × 10^0^	1.55%
1	−1.172 × 10^−1^	5.756 × 10^−1^	−1.189 × 10^1^	
2	1.024 × 10^−2^	−2.990 × 10^−1^	−3.963 × 10^1^	
3	−1.768 × 10^−4^	3.046 × 10^−3^	−1.894 × 10^1^	
4	9.452 × 10^−7^	−8.957 × 10^−6^	−4.335 × 10^0^	

**Table 7:** Regression parameters for CaCl_2_-LiNO_3_-KNO_3_/H_2_O and AARD value.

*i*	*A_i_*	*B_i_*	*C_i_*	AARD
0	−2.262 × 10^−2^	−3.844 × 10^−1^	5.668 × 10^−1^	2.34%
1	5.616 × 10^−1^	1.184 × 10^0^	1.367 × 10^0^	
2	−2.523 × 10^−2^	−1.498 × 10^−2^	5.033 × 10^0^	
3	4.274 × 10^−4^	−3.524 × 10^−3^	−3.042 × 10^1^	
4	−2.421 × 10^−6^	2.944 × 10^−5^	−1.620 × 10^1^	

**Table 8:** *p* of CaCl_2_-LiNO_3_-KNO_3_(15.5:5:1)/H_2_O.

*w*/wt.%		Saturated Vapor Pressure *p* (kPa) at Each Temperature *T* (°C)								
50.0	*T*	20.1	25.7	30.2	35.3	40.6	45.0	50.7	55.4	60.0
	*p*	0.634	0.901	1.225	1.705	2.371	3.064	4.325	5.626	7.206
	*T*	65.2	70.7	75.0	80.9	85.7	90.0	95.2	100.0	
	*p*	9.336	12.075	14.800	19.100	23.536	27.931	33.519	40.578	
55.0	*T*	21.2	28.0	32.1	35.0	39.9	45.1	52.0	55.6	60.0
	*p*	0.443	0.712	0.939	1.148	1.569	2.210	3.407	4.249	5.387
	*T*	65.2	70.2	75.0	80.9	85.0	90.4	95.0	100.0	
	*p*	7.060	9.016	11.301	14.825	17.699	21.912	26.011	31.898	
60.0	*T*	21.0	26.1	30.0	35.0	41.0	45.0	50.0	55.1	60.4
	*p*	0.280	0.412	0.541	0.769	1.166	1.492	2.071	2.855	4.026
	*T*	65.5	70.2	75.0	80.0	85.4	90.4	95.2	100.1	
	*p*	5.355	6.901	8.873	11.106	14.063	17.268	21.176	25.912	
65.0	*T*	25.1	30.0	35.0	40.5	45.0	50.0	55.6	60.5	65.6
	*p*	0.226	0.341	0.490	0.744	0.985	1.339	1.958	2.675	3.665
	*T*	71.1	75.4	80.1	85.2	90.0	94.6	100.5		
	*p*	4.971	6.282	7.749	9.894	12.395	15.241	19.243		

**Table 9:** *ρ* of CaCl_2_-LiNO_3_-KNO_3_(15.5:5:1)/H_2_O.

*w*/wt.%		Density *ρ* (g·cm^−3^) at Each Temperature *T* (°C)								
50.0	*T*	20.0	30.0	40.0	50.0	60.0	70.0	80.0	90.0	100.0
	*ρ*	1.4614	1.4563	1.4488	1.4412	1.4352	1.4278	1.4215	1.4141	1.4067
55.0	*T*	20.0	30.0	40.0	50.0	60.0	70.0	80.0	90.0	100.0
	*ρ*	1.5181	1.5111	1.5042	1.4977	1.4906	1.4834	1.4761	1.4696	1.4618
60.0	*T*	20.0	30.0	40.0	50.0	60.0	70.0	80.0	90.0	100.0
	*ρ*	1.5784	1.5713	1.5642	1.5547	1.5495	1.5420	1.5351	1.5287	1.5229
65.0	*T*			40.0	50.0	60.0	70.0	80.0	90.0	100.0
	*ρ*			1.6245	1.6185	1.6131	1.6058	1.5991	1.5915	1.5820

**Table 10:** *η* of CaCl_2_-LiNO_3_-KNO_3_(15.5:5:1)/H_2_O.

*w*/wt.%		Viscosity *η* (mPa·s) at Each Temperature *T* (°C)								
50.0	*T*	20.0	30.0	40.0	50.0	60.0	70.0	80.0	90.0	100.0
	*H*	20.13	14.51	10.56	8.00	5.98	4.77	4..11	3.47	2.95
55.0	*T*	20.0	30.0	40.0	50.0	60.0	70.0	80.0	90.0	100.0
	*H*	45.51	29.57	21.63	15.40	11.42	7.79	6.19	5.18	4.35
60.0	*T*	20.0	30.0	40.0	50.0	60.0	70.0	80.0	90.0	100.0
	*H*	144.05	81.88	51.66	33.48	24.07	16.91	12.51	9.47	7.71
65.0	*T*			40.0	50.0	60.0	70.0	80.0	90.0	100.0
	*H*			144.90	80.97	52.26	37.07	25.91	19.38	14.55

**Table 11:** *C_p_* of CaCl_2_-LiNO_3_-KNO_3_(15.5:5:1)/H_2_O.

*w*/wt.%			Specific Heat Capacity *C_p_* (kJ·kg^−1^·K ^−1^) at Each Temperature *T* (°C)								
50.0	*T*	10.0	20.0	30.0	40.0	50.0	60.0	70.0	80.0	90.0	100.0
	*C_p_*	2.438	2.444	2.458	2.473	2.487	2.501	2.518	2.527	2.542	2.563
55.0	*T*	10.0	20.0	30.0	40.0	50.0	60.0	70.0	80.0	90.0	100.0
	*C_p_*	2.313	2.321	2.335	2.350	2.366	2.380	2.400	2.417	2.432	2.452
60.0	*T*	10.0	20.0	30.0	40.0	50.0	60.0	70.0	80.0	90.0	100.0
	*C_p_*	2.189	2.193	2.206	2.216	2.227	2.241	2.254	2.266	2.286	2.303
65.0	*T*	10.0	20.0	30.0	40.0	50.0	60.0	70.0	80.0	90.0	100.0
	*C_p_*	2.039	2.045	2.057	2.071	2.085	2.098	2.110	2.127	2.130	2.146

**Table 12:** *C_p_* of solid KNO_3_ at atmosphere pressure.

Reagent		Specific Heat Capacity *C_p_* (kJ·kg^−1^·K^−1^) at Each Temperature *T* (°C)									
KNO_3_	*T*	10.0	20.0	30.0	40.0	50.0	60.0	70.0	80.0	90.0	100.0
	*C_p_*	0.930	0.952	0.968	0.974	0.988	1.040	1.053	1.072	1.090	1.119

**Table 13:** Δ*H*_mix_ of CaCl_2_-LiNO_3_-KNO_3_(15.5:5:1) /H_2_O at 25.0 °C.

*w*/wt.%	50.0	55.0	60.0	65.0
Δ*H*_mix_/kJ·kg^−1^	149.310	150.501	150.275	151.292

**Table 14:** *h* of CaCl_2_-LiNO_3_-KNO_3_(15.5:5:1)/H_2_O.

*w*/wt.%		Specific Enthalpy *h* (kJ·kg^−1^) at Each Temperature *T* (°C)									
50.0	*T*	10.0	20.0	30.0	40.0	50.0	60.0	70.0	80.0	90.0	100.0
	*h*	296.787	321.185	345.720	370.395	395.214	420.183	445.305	470.584	496.025	521.632
55.0	*T*	10.0	20.0	30.0	40.0	50.0	60.0	70.0	80.0	90.0	100.0
	*h*	293.028	316.239	339.568	363.026	386.622	410.368	434.272	458.345	482.598	507.039
60.0	*T*	10.0	20.0	30.0	40.0	50.0	60.0	70.0	80.0	90.0	100.0
	*h*	290.874	312.767	334.776	356.907	379.172	401.578	424.134	446.849	469.733	492.793
65.0	*T*	10.0	20.0	30.0	40.0	50.0	60.0	70.0	80.0	90.0	100.0
	*h*	287.666	308.113	328.685	349.382	370.205	391.155	412.233	433.438	454.773	476.237

**Table 15:** *s* of CaCl_2_-LiNO_3_-KNO_3_(15.5:5:1)/H_2_O.

*w*/wt.%			Entropy *s* (kJ·kg^−1^·K^−1^) at Each Temperature *T* (°C)								
50.0	*T*	10.0	20.0	30.0	40.0	50.0	60.0	70.0	80.0	90.0	100.0
	*s*	1.412	1.496	1.578	1.658	1.736	1.813	1.889	1.963	2.036	2.108
55.0	*T*	10.0	20.0	30.0	40.0	50.0	60.0	70.0	80.0	90.0	100.0
	*s*	1.417	1.498	1.576	1.652	1.727	1.800	1.872	1.943	2.014	2.083
60.0	*T*	10.0	20.0	30.0	40.0	50.0	60.0	70.0	80.0	90.0	100.0
	*s*	1.422	1.500	1.574	1.645	1.716	1.785	1.852	1.919	1.985	2.050
65.0	*T*	10.0	20.0	30.0	40.0	50.0	60.0	70.0	80.0	90.0	100.0
	*s*	1.431	1.502	1.571	1.638	1.704	1.769	1.832	1.895	1.955	2.016

**Table 16:** Concentration and generation temperature for different working pairs.

Working Pair	CaCl_2_-LiNO_3_- KNO_3_ (15.5:5:1)/H_2_O	LiBr/ H_2_O	CaCl_2_-LiBr-LiNO_3_- KNO_3_(16.2:2:2:1) /H_2_O	CaCl_2_-LiNO_3_- LiBr (8.72:1:1)/H_2_O	CaCl_2_-LiBr (1.35:1) /H_2_O
Dilute solution/wt.%	60.5	56.4	58.5	57.3	55.8
Strong solution/wt.%	63.5	59.4	61.5	60.3	58.8
Generation temperature/°C	74.0	81.0	74.8	73.3	74.8

**Table 17:** State parameters of streams in the cycle with CaCl_2_-LiNO_3_-KNO_3_(15.5:5:1)/H_2_O.

Point	Stream	*p*/kPa	*T*/°C	*w*/wt.%	*h*/kJ·kg^−1^	*D*/kg·s^−1^	*s*/kJ·kg^−1^·K^−1^
1	Water	0.872	5.0	0	439.6	1.0	0.07621
1ʹ	Vapor	0.872	5.0	0	2927.9	1.0	9.02690
2	Dilute solution	0.872	37.0	60.5	349.2	21.2	0.09149
3	Water	6.290	37.0	0	573.5	1.0	0.53190
4	Strong solution	6.290	74.0	63.5	424.4	20.2	0.33201
4ʹ	Vapor	6.290	74.0	0	3054.2	1.0	8.5368
5	Dilute solution	6.290	69.2	60.5	420.9	21.2	0.31351
6	Strong solution	0.872	41.0	63.5	353.7	20.2	0.11525
7	Dilute solution	--	63.9	60.5	409.9	21.2	0.27782
8	Strong solution	--	44.3	63.5	360.7	20.2	0.13754

**Table 18:** Coefficient of performance (COP) of the cycle with different working pairs.

Working Pair	CaCl_2_-LiNO_3_-KNO_3_ (15.5:5:1) /H_2_O	LiBr /H_2_O	CaCl_2_-LiBr-LiNO_3_-KNO_3_ (16.2:2:2:1) /H_2_O	CaCl_2_-LiNO_3_-LiBr (8.72:1:1) /H_2_O	CaCl_2_-LiBr (1.35:1) /H_2_O
*COP*	0.801	0.762	0.793	0.805	0.788

**Table 19:** The exergy destruction in each part of absorption refrigeration cycle using CaCl_2_-LiNO_3_-KNO_3_(15.5:5:1)/H_2_O and LiBr/H_2_O.

Part	Exergy Destruction/kW	
	CaCl_2_-LiNO_3_-KNO_3_ (15.5:5:1)/H_2_O	LiBr/H_2_O
Evaporator	0.5	0.5
Condenser	0.4	0.8
Absorber	52.1	55.3
Generator	282.6	332.7
Heat exchanger	25.5	31.4

## Nomenclature

*T*	temperature, °C
*w*	mass concentration, %
*T_C_*	crystallization temperature, °C
*p*	saturated vapor pressure, kPa
*ρ*	density, g·cm^−3^
*η*	dynamic viscosity, mPa·s
*C*_p_	specific heat capacity, kJ·kg^−1^·K^−1^
Δ*H*_mix_	dissolution enthalpy, kJ·kg^−1^
*h*	specific enthalpy, kJ·kg^−1^
*s*	specific entropy, kJ·kg^−1^·K^−1^
AARD	average absolute relative deviation
*a*	circulation ratio
*COP*	coefficient of performance
*E*	exergy
Δ*E*	exergy destrction
*η_E_*	exergy efficiency
*R_C_*	corrosion rate, μm∙y^−1^
